# Genetic differentiation in East African ethnicities and its relationship with endurance running success

**DOI:** 10.1371/journal.pone.0265625

**Published:** 2022-05-19

**Authors:** André L. S. Zani, Mateus H. Gouveia, Marla M. Aquino, Rodrigo Quevedo, Rodrigo L. Menezes, Charles Rotimi, Gerald O. Lwande, Collins Ouma, Ephrem Mekonnen, Nelson J. R. Fagundes

**Affiliations:** 1 Postgraduate Program in Genetics and Molecular Biology, Institute of Biosciences, Federal University of Rio Grande do Sul, Porto Alegre, RS, Brazil; 2 Center for Research on Genomics and Global Health, National Human Genome Research Institute, National Institutes of Health, Bethesda, Maryland, United States of America; 3 Department of Genetics, Ecology and Evolution, Institute of Biological Sciences, Federal University of Minas Gerais, Belo Horizonte, MG, Brazil; 4 School of Physical Education, Physical Therapy and Dance, Federal University of Rio Grande do Sul, Porto Alegre, RS, Brazil; 5 Department of Biomedical Sciences and Technology, Maseno University, Maseno, Kenya; 6 Institute of Biotechnology, Addis Ababa University, Addis Ababa, Ethiopia; 7 Postgraduate Program in Animal Biology, Institute of Biosciences, Federal University of Rio Grande do Sul, Porto Alegre, RS, Brazil; McMaster University, CANADA

## Abstract

Since the 1960s, East African athletes, mainly from Kenya and Ethiopia, have dominated long-distance running events in both the male and female categories. Further demographic studies have shown that two ethnic groups are overrepresented among elite endurance runners in each of these countries: the Kalenjin, from Kenya, and the Oromo, from Ethiopia, raising the possibility that this dominance results from genetic or/and cultural factors. However, looking at the life history of these athletes or at loci previously associated with endurance athletic performance, no compelling explanation has emerged. Here, we used a population approach to identify peaks of genetic differentiation for these two ethnicities and compared the list of genes close to these regions with a list, manually curated by us, of genes that have been associated with traits possibly relevant to endurance running in GWAS studies, and found a significant enrichment in both populations (Kalenjin, *P* = 0.048, and Oromo, *P* = 1.6x10^-5^). Those traits are mainly related to anthropometry, circulatory and respiratory systems, energy metabolism, and calcium homeostasis. Our results reinforce the notion that endurance running is a systemic activity with a complex genetic architecture, and indicate new candidate genes for future studies. Finally, we argue that a deterministic relationship between genetics and sports must be avoided, as it is both scientifically incorrect and prone to reinforcing population (racial) stereotyping.

## Introduction

**Fig 1:**
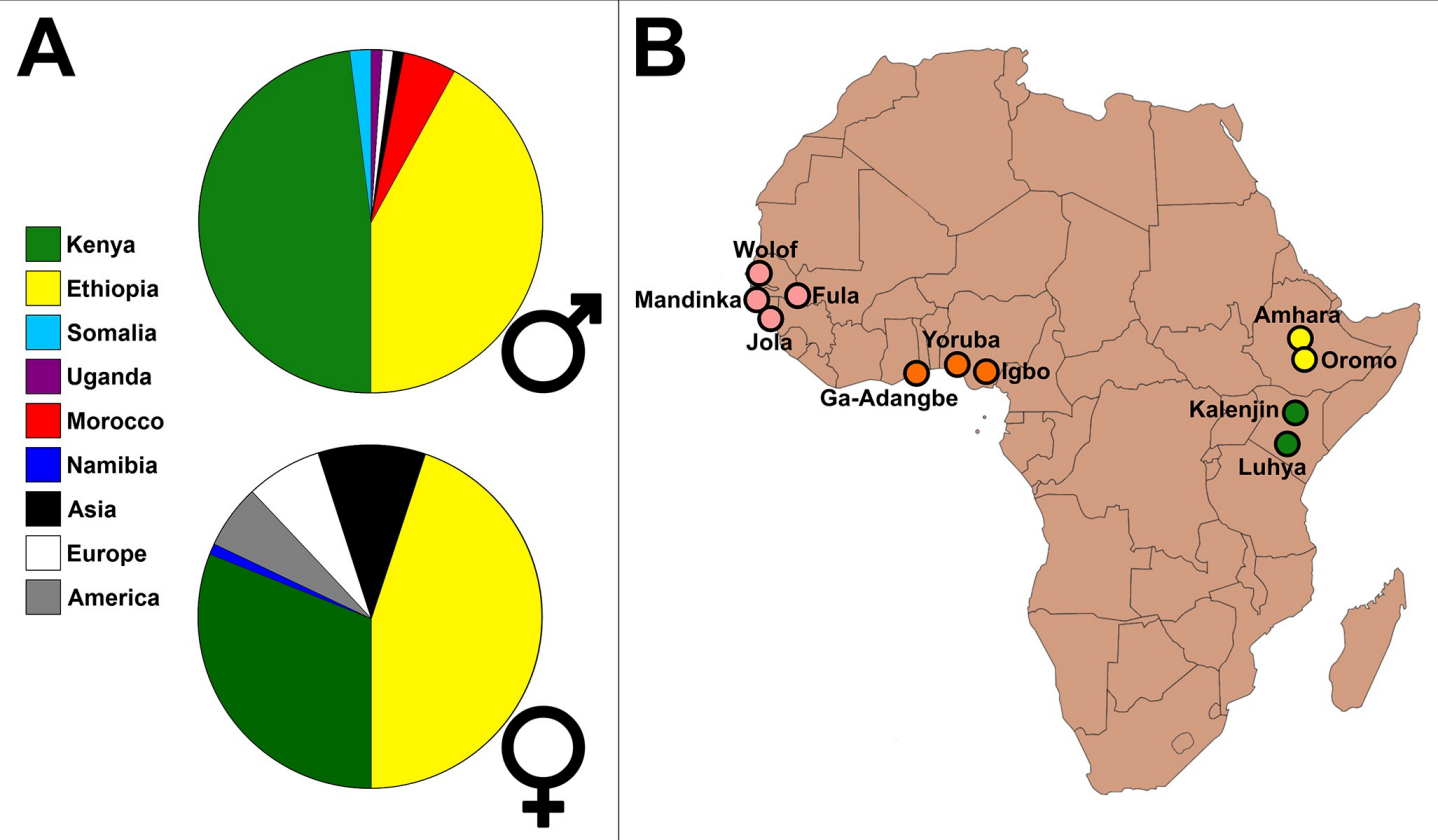
**A)** Place of birth of the 100 best marathon runners of all time, men and women, as listed by the International Association of Athletics Federations on June 29, 2019. Note the large fraction corresponding to Kenya and Ethiopia, in both sexes. **B)** Approximate locations for the African ethnolinguistic groups (populations) used in this study.

The ability to run long distances has played an important role in human evolution, and our species stands among the best endurance runners of all mammals [1]. In modern times, such ability manifests itself in athletics, especially in long-distance running events, in which elite athletes can run a marathon (42.195 km) in about two hours. East African athletes have dominated these events since the 1960s, in both the male and female categories [2]. Their overrepresentation among the world’s best endurance runners is so impressive that, as of January 2021, of the 100 athletes from each sex holding the best marathon times in history, 76 women and 93 men were born in East Africa (https://www.worldathletics.org/disciplines/road-running/marathon) (Fig 1A). The vast majority of these athletes come from two countries and, more importantly, two ethnicities within those countries: the Kalenjin, from Kenya, and the Oromo, from Ethiopia [2,3]. More information about these countries and ethnicities can be found in the *S1 Text*.

While the regional and ethnical clustering of best-ever performing athletes may suggest a shared genetic or cultural background as important factors, no simple explanation for their dominance has emerged yet [4]. This is not surprising, considering that the success in a systemic activity such as endurance running is probably the result of a complex interplay between multiple innate and trainable traits [4]. Environmental factors suggested as relevant to the success of Kenyan and Ethiopian athletes include: (1) a favorable diet; (2) living and training at high altitude; (3) socio-economic motivation to escape poverty; and (4) the habit of running to school as children [5]. However, none of these factors alone can explain such success, as they are also present in many countries and populations that have never produced world-class athletes in this proportion [4]. Besides, even in East African populations, evidence for a causal relation in each of those cases is, at best, tenuous [5]. This suggests that either many environmental factors act in concert to favor endurance running success, that there is an innate component that needs to be accounted for, or, more likely, both.

Regarding genetic factors, only two candidate genes have been investigated in East African populations [4,5]. Yang et al. [6] studied the ACTN3 gene in both Kenyan and Ethiopian athletes while Scott et al. [7] and Ash et al. [8] investigated the ACE gene in Kenyan and Ethiopian athletes, respectively. In those three studies, the frequency of a favorable allele discovered in Eurasian runners was compared between endurance athletes and controls from their respective countries. None of them found a statistically significant difference between athletes and controls [6–8]. Similarly, differences in mitochondrial haplogroup composition have been investigated in both Ethiopian and Kenyan endurance runners under the same case-control design. While no differences have been found in Ethiopia [9], Kenyan athletes exhibited a higher frequency of haplogroup L0 and a lower frequency of haplogroup L3 [10], but this result could be due to population stratification if cases and controls do not come from the same ethnicities, for example. More recently, cohorts of athletes and controls from Kenya and Ethiopia have been used to validate 45 markers that were preliminarily associated with endurance running in a Genome-wide Association Study (GWAS) [11]. Six markers showed differences between Kenyan athletes and controls, while three different markers showed differences between Ethiopian athletes and controls. The authors conclude, also based on the results of other cohorts, that there is no evidence for a common genetic profile specific to endurance athletes [11].

Tucker *et al*. [4] criticized the candidate gene and case-control approaches previously applied to these populations, especially when studying highly complex traits like endurance performance. They argued that a case-control design will fail if both groups share a similar genetic favorable background but differ in training history. Instead, they proposed that the over-representation of Kalenjin and Oromo in long-distance running events should be more easily understood as a populational phenomenon. That is, these populations would have a higher frequency of many favorable alleles, which would allow the appearance of individuals with multiple favorable genotypes through the genome much more frequently than in other populations. When exposed to the right environmental conditions (including training), present in those countries, these individuals would then be able to reach the elite endurance runner status, accounting for the overrepresentation of East Africans among those athletes.

**Fig 2:**
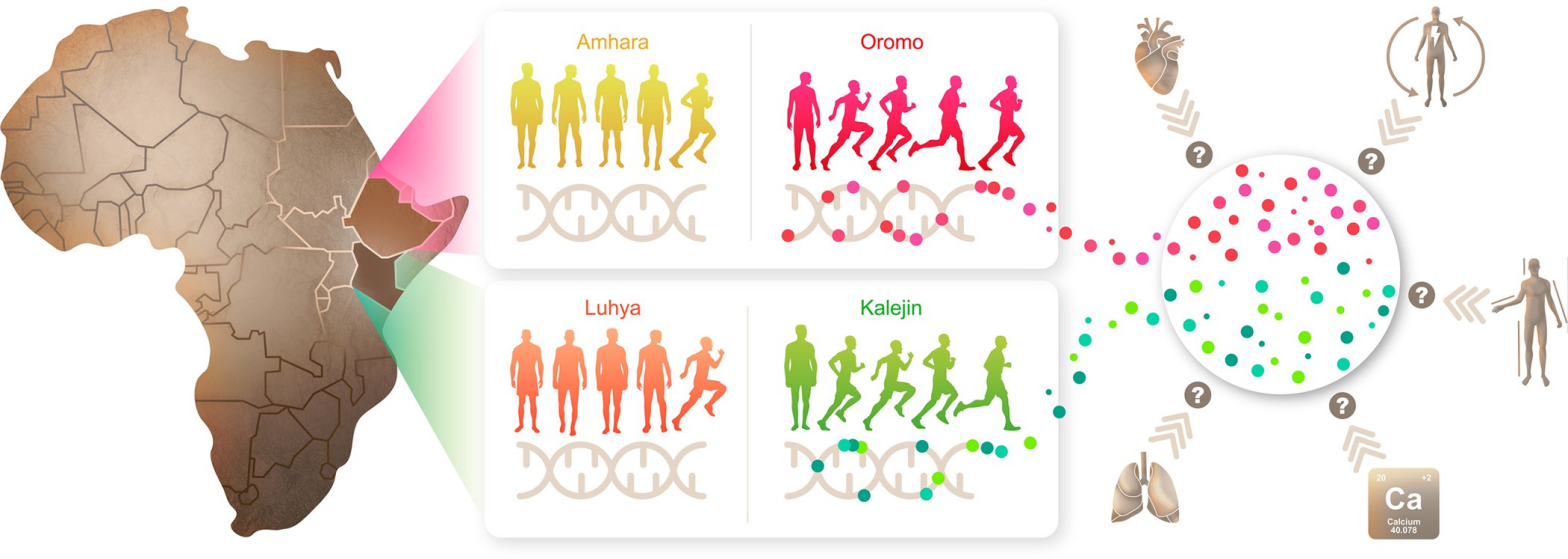
Overview of the study design. For each country, Ethiopian and Kenya, ethnicities overrepresented among endurance running athletes, Oromo and Kalenjin, respectively, were compared to populations that are underrepresented among athletes, Amhara and Luhya, respectively. Note that all populations may contain individuals that are genetically predisposed to endurance running or not, though in different proportions. Population comparisons allowed the identification of genome regions highly differentiated in Oromo and Kalenjin and the genes in their vicinity. The list of genes was then interrogated for enrichment for phenotypic traits that may be relevant for endurance running, such as heart function, energy metabolism, anthropometric traits, calcium homeostasis, and lung function, among others. Please see the Methods for a detailed description of all steps performed during the study.

In the present study, we followed this reasoning by comparing genomic populational data among different ethnicities in East Africa. We hypothesize that the alleles that predispose to endurance running should be common in the general population of these ethnic groups and, consequently, that genetic factors associated with athletic success in Kalenjin and Oromo should be, at least partially, close to the genomic regions of greater differentiation in these ethnicities, enabling the identification of molecular processes that contribute to long-distance running (Fig 2). This approach has the great advantage of not relying on sampling athletes. By treating a phenotype (in this study, endurance running capacity) as a populational characteristic we can use data from public genomic datasets of genomic variation to test our hypothesis, similar to studies about the genetic basis of adaptation to high-altitude environments, for example [12,13].

## Results

**Fig 3:**
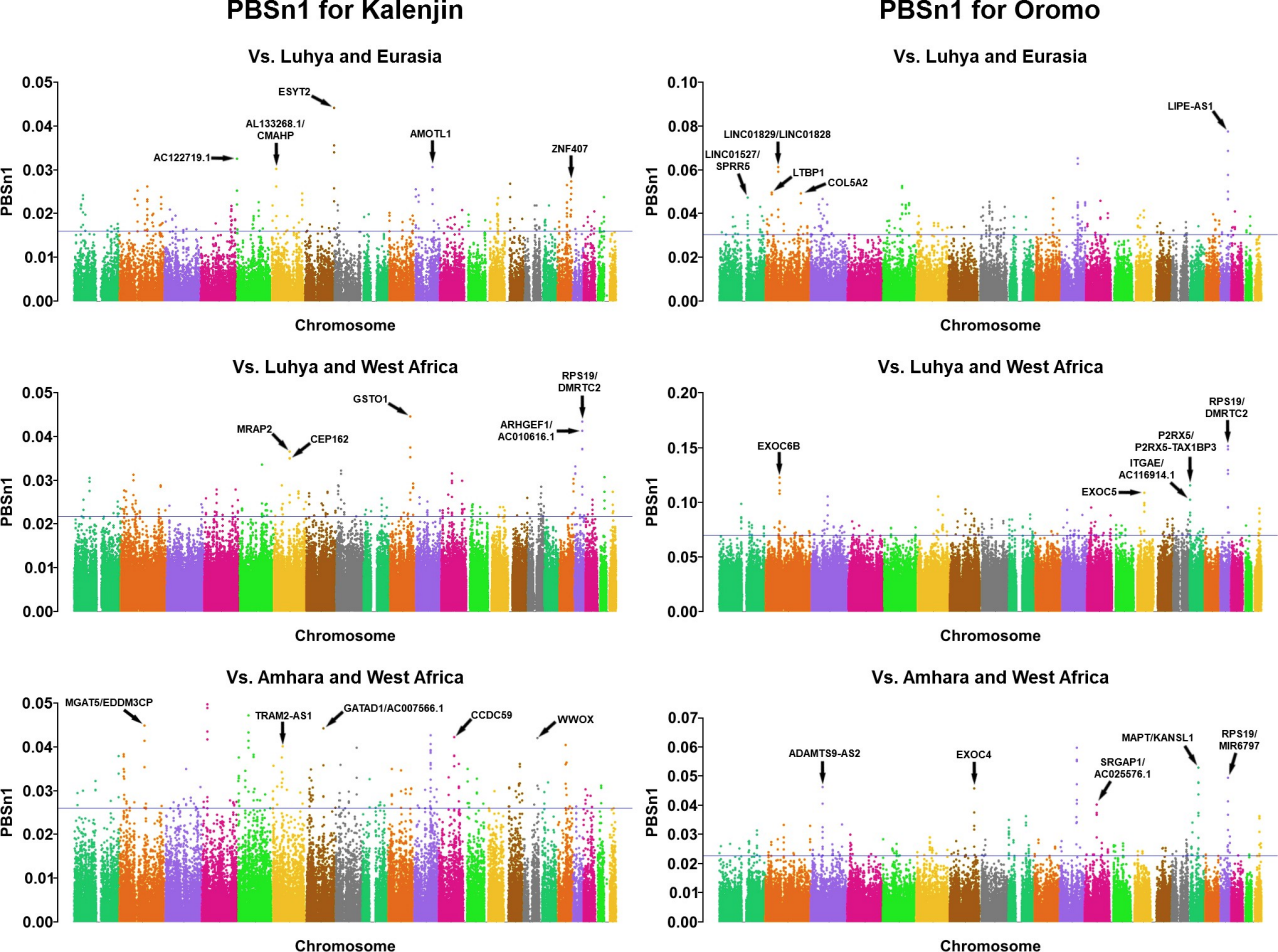
Manhattan plots of PBSn1 values for Kalenjin and Oromo considering all comparisons (see *Material and Methods* for details). Each dot represents a window of 20 SNPs. The blue line indicates the 0.1% highest values. Note the different scale among comparisons. Genes associated with the five non-intergenic windows with the highest PBSn1 are shown. For the complete list of genes, see S1 Dataset.

Using the normalized Population Branch Statistic (PBSn1) [14], we identified, for Kalenjin and Oromo, respectively, 297 and 352 genes linked to highly differentiated genomic regions (Fig 3; S1 Dataset). We submitted these lists to an enrichment analysis [15] to recover phenotypes or traits showing enriched gene-sets. After Benjamini-Hochberg false discovery rate (FDR) correction for multiple comparison, this resulted in 47 and 97 trait-related gene-sets enriched for Kalenjin and Oromo, respectively (S2 Dataset). When compared to a list of 628 endurance-relevant gene-sets (S3 Dataset) selected *a priori* from the GWAS catalog database [16], we found 10 matches for Kalenjin (Table 1) and 27 for Oromo (Table 2) (one-tailed Fisher’s exact test *P* = 0.048, odds ratio (OR) = 2.003, and *P* = 1.6x10^-5^, OR = 2.918, respectively; see S1 Table for a comparison of traits between these populations). When the closely related populations were considered (see *Material and Methods* for details), the Luhya, from Kenya, showed no significant enrichment (*P* = 0.341; OR = 1.840; 2 traits), while the Amhara, from Ethiopia, were enriched for endurance-relevant traits (*P* = 0.011; OR = 3.122; 8 traits). However, in both cases, these populations had, respectively, fewer endurance-relevant traits than its national counterparts (one-tailed Fisher’s exact test: Luhya vs. Kalenjin, *P* = 0.019, OR = 5.059; Amhara vs. Oromo, *P* = 8.1x10^-4^, OR = 3.479).

**Table 1:** Enriched gene-sets, in Kalenjin, for “endurance relevant” traits in GWAS Catalog (*P*<0.05 after FDR correction). The number of genes in Kalenjin’s list is shown for each trait (Genes in set). Gene’s names can be found in S2 Dataset.

Kalenjin			
GWAS catalog gene-set	Genes in set	Genes in set (total)	*P*-value
**Heel bone mineral density**	19	834	<0.001
**Waist-to-hip ratio adjusted for BMI**	9	350	0.025
**Hip shape (DXA scan)**	2	6	0.031
**Subcutaneous adipose tissue**	4	62	0.031
**FEV1**	6	175	0.033
**LDL cholesterol**	6	181	0.033
**Lung function (FEV1/FVC)**	6	184	0.034
**Glucose homeostasis traits**	4	73	0.037
**Waist circumference**	4	73	0.037
**Height**	10	529	0.044

FEV1 = Forced expiratory volume in 1 second.

DXA = Dual-energy X-ray absorptiometry.

LDL = Low-density lipoprotein.

FVC = Forced vital capacity.

BMI = Body mass index.

**Table 2:** Enriched gene-sets, in Oromo, for “endurance relevant” traits in GWAS Catalog (*P*<0.05 after FDR correction). The number of genes in Oromo’s list is shown for each trait (Genes in set). Gene’s names can be found in S2 Dataset.

Oromo			
GWAS catalog gene-set	Genes in set	Genes in set (total)	*P*-value
**Diastolic blood pressure**	22	648	<0.001
**Hand grip strength**	9	156	<0.001
**Pulse pressure**	18	690	<0.001
**Systolic blood pressure**	19	783	0.001
**Body mass index**	24	1197	0.001
**Waist circumference**	6	73	0.002
**Height**	14	529	0.003
**Lung function (FVC)**	8	178	0.004
**Waist-to-hip ratio adjusted for BMI**	11	350	0.004
**Lung function (FEV1)**	5	65	0.006
**Peak expiratory flow**	6	115	0.009
**Waist circumference adjusted for BMI in active individuals**	5	83	0.014
**Body fat distribution (arm fat ratio)**	6	129	0.014
**Atrial fibrillation**	8	238	0.014
**Waist circumference adjusted for BMI (joint analysis main effects and physical activity interaction)**	5	85	0.014
**Lung function (FEV1/FVC)**	7	184	0.015
**Bone mineral density (hip)**	4	55	0.021
**Hypertension**	5	100	0.023
**Hip circumference adjusted for BMI**	5	101	0.024
**Waist-hip ratio**	4	60	0.025
**Lean body mass**	2	7	0.028
**Heel bone mineral density**	15	834	0.032
**Vigorous physical activity**	2	8	0.032
**Waist-to-hip ratio adjusted for body mass index**	5	114	0.034
**FEV1**	6	175	0.036
**Hip circumference**	4	73	0.039
**Waist circumference adjusted for body mass index**	5	126	0.044

FEV1 = Forced expiratory volume in 1 second.

FVC = Forced vital capacity.

BMI = Body mass index.

Thus, genes close to highly differentiated genomic regions are enriched for endurance-relevant traits in Kalenjin and Oromo, and both have more endurance-relevant gene-sets than its national pairs. The 10 endurance-relevant gene-sets for Kalenjin and the 27 for Oromo comprised 49 and 96 candidate genes (S2 Dataset). For each population comparison (see *Material and Methods* for details), we also selected the genes in the five most differentiated genomic regions, highlighting seven candidate genes for Kalenjin: *TRAM2-AS1*, *WWOX*, *ARHGEF1*, *GATAD1*, *AMOTL1*, *GSTO1*, and *MRAP2*; and eight for Oromo: *LTBP1*, *COL5A2*, *MIR6797*, *ADAMTS9-AS2*, *EXOC4*, *EXOC5*, *EXOC6B*, and *LIPE-AS1* whose biological function is related to the endurance-relevant gene-sets. These functions affect anthropometric/biomechanical traits (*LTBP1*, *TRAM2-AS1*, *COL5A2*, *WWOX* and *MIR6797*), lung function (*WWOX*), blood pressure and the circulatory system (*ADAMTS9-AS2*, *ARHGEF1*, *GATAD1*, *AMOTL1*, and *GSTO1*), glucose metabolism (*EXOC4*, *EXOC5*, *EXOC6B* and *WWOX*), fatty acids metabolism (*MRAP2* and *LIPE-AS1*) and calcium homeostasis (*WWOX*, *MIR6797*, *MRAP2*, *GSTO1*).

## Discussion

In this study, we used publicly available genomic data to tackle the hypothesis that genetic variation could be associated with endurance running in East African populations [4], but there are limitations. Our approach identified highly differentiated genomic regions irrespective of whether they are relevant to endurance running or not. Thus, we had to restrict our analysis to traits whose relationship with endurance running is well documented. Given the widespread pleiotropy affecting complex phenotypes, meaningful biological associations may have gone unnoticed. On the other hand, further studies are also necessary to corroborate and clarify the specific association between these candidate genes and endurance running in different human populations. While many of the biological associations discussed below may look speculative, they serve as a starting point for investigating the possible contribution of these genes to endurance running.

There was an abundance of anthropometric-related traits, such as height, waist circumference, and waist-to-hip ratio, among the enriched sets for both Kalenjin and Oromo. Eksterowicz *et al*. [17] found that Marathon finishing-time was positively correlated with upper limb length, torso length, hip width, and waist-hip ratio in a sample of Kenyan endurance runners. More generally, anthropometric features affect stride length, movement stability, air and ground resistance [1,17,18]. *LTBP1*, one of the most differentiated genes for Oromo, participates in the molecular pathway associated with those phenotypes. LTBP1 binds to TGF-ß1, facilitating its export to the extracellular matrix of bone cells, where it plays a key role in chondrocyte maturation, mineralization, and bone remodeling [19,20].

The structure of bones and tendons also plays an important role in physical activity, including long-distance running. Less flexibility and greater stiffness are observed in the lower limb’s tendons of long-distance runners [21,22], and lower overall body flexibility is associated with running economy by increasing body stability and enhancing the use of elastic energy [23]. Two genes highly differentiated in Kalenjin and Oromo: *TRAM2-AS1* and *COL5A2*, respectively, are associated with the synthesis and organization of type I collagen, the main protein in ligaments and tendons [24]. *TRAM2-AS1* encodes an antisense RNA against *TRAM2*, which participates in type I collagen’s biosynthesis [25]. Given the importance of antisense RNAs in gene expression regulation [26], *TRAM2-AS1* may modulate the synthesis of type I collagen. Likewise, *COL5A2* encodes, together with *COL5A1* and *COL5A3*, one of the three alpha chains that form type V collagen, which on its turn regulates the assembly and structure of type I collagen fibrils [27,28]. Mutations in *COL5A2* or *COL5A1* account for over 90% of the cases of the classic Ehlers-Danlos syndrome, characteristic for joint hypermobility [29]. Two polymorphisms in *COL5A1* (rs12722, C/T, and rs71746744, -/AGGG) have been directly associated with performance in long-distance running, with individuals carrying the T/T or AGGG/AGGG genotypes being considerably faster and less flexible [30,31]. Variants in *COL5A2* may have a similar effect to *COL5A1*, considering that these genes encode different subunits of the same protein and that mutations in both have the same clinical outcome [29].

Long-distance running has also been associated with bone mineral density, which is increased in the legs and reduced in the vertebrae of runners compared to controls [32–34]. We found enriched gene-sets related to bone mineral density for both populations. In addition to *LTBP1*, previously discussed, other highly differentiated genes that could affect this trait are *WWOX*, shared by both populations, *MIR6797* in Oromo, and *TRAM2-AS1* in Kalenjin. The products encoded by these genes interact with the transcription factor RUNX2, the major regulator of bone development [35]. The first two act as negative regulators of *RUNX2* expression, controlling osteoblast differentiation [36,37]. Indeed, WWOX deficient mice show a considerable delay in skeletal development and mineralization, including reduced blood calcium levels [36]. RUNX2 also regulates the expression of *TRAM2* in bone cells, affecting the availability of type I collagen in the bone extracellular matrix [38].

Together with anthropometric traits, physiological processes are central to endurance running [39]. In general, good endurance runners will have a high capacity to consume oxygen (high maximum oxygen uptake—VO_2_max, high capacity to mobilize oxygen and energy), but will consume less oxygen to run at intermediate speeds (good running economy, low energy requirement) [23]. The efficiency of the aerobic metabolism required for maintaining high intensity physical activity depends on several distinct physiological steps: absorbing oxygen from the air, transporting it through the bloodstream to the skeletal muscle, mobilizing energy reserves, and performing muscle contraction [23]. Lung function, representing the first step of this pathway has been correlated with performance in endurance running trials [40,41], and was improved in rats artificially selected for endurance running performance [42], as expected from the considerably high heritability shown by VO_2_max (*h*^*2*^ ≅ 0.56) [43]. We found enriched gene-sets for lung function in both Kalenjin and Oromo. *WWOX*, present in the gene lists of both populations, has been associated with lung function in GWAS [44,45], case-control, and family-based studies [46], even though the molecular mechanism is not fully understood.

The next step, oxygen transport, is highly dependent on blood pressure (BP), on the amount of available hemoglobin, and tissue vascularization. Diastolic BP has been identified as a predictor of endurance performance [47,48], and endurance runners frequently have left ventricular hypertrophy [49]. Oromo shows enriched gene-sets related to BP and cardiac conduction. One of its highly differentiated genes, *ADAMTS9-AS2*, encodes the antisense RNA of the *ADAMTS9*, which codes for a metalloproteinase essential for the normal development and homeostasis of the heart and arteries [50]. Unlike Oromo, Kalenjin does not have enriched gene-sets associated with BP. However, four of its highly differentiated genes (*ARHGEF1*, *GATAD1*, *AMOTL1*, and *GSTO1*) participate in biological processes related to BP. *ARHGEF1* is activated by angiotensin II in arterial smooth muscle cells, leading to increased BP [51]. *ACE* codes for the enzyme that produces angiotensin II, and has been associated with endurance running success in other studies [52], though not in Ethiopian runners [8]. *GATAD1* and *AMOTL1* seem to be more important in cardiac muscle development, but may affect BP indirectly. *GATAD1* is expressed in ventricular myocytes, and mutations in this gene cause dilated cardiomyopathy, a disease characterized by excessive enlargement of cardiac ventricles [53]. *AMOTL1* has been associated with the enlargement and proliferation of cardiomyocytes, cardiac hypertrophy [54], and angiogenesis [55,56]. Finally, *GSTO1* downregulates the activity of the cardiac ryanodine channel RYR2, which releases calcium from the sarcoplasmic reticulum into the cytoplasm to perform muscle contraction [57]. Interestingly, *RYR2* is in the Oromo gene list—in the gene sets associated with lung function (S2 Dataset), and has been associated with increased VO_2_max trainability in Europeans [58].

Most metabolic energy for endurance running comes from aerobic glycolysis in the mitochondria [59]. Both Kalenjin and Oromo have enriched gene-sets associated with glucose metabolism: “Glucose homeostasis traits” and “Vigorous physical activity”, respectively. In the Oromo, three genes encoding proteins of the exocyst complex were linked to high differentiation regions (*EXOC4*, *EXOC5*, and *EXOC6B*). The exocyst is essential in the insulin-induced transport to the cell membrane of the main glucose transporter (GLUT4) in the muscle (skeletal and cardiac) and adipose tissue [60,61]. The knockout of either of those genes reduces the entry of glucose into adipocytes and skeletal muscle cells considerably [60–62]. Within the cell, WWOX binds to HIF1α and modulates aerobic glycolysis by inhibiting the activation of genes that induce aerobic metabolism. *WWOX* deficient cells show an increase in HIF1α levels and activity, as well as an increase in glucose uptake [63]. *HIF1A*, the gene encoding HIF1α, has already been directly associated with elite long-distance runner status [64].

On the other hand, the use of fatty acids in energy metabolism is linked to the ability to maintain physical activity for a longer period while preserving the systemic glucose levels [65]. Again, both populations had lipid metabolism enriched gene-sets. In the Oromo list, *LIPE-AS1* encodes the antisense RNA for *LIPE*, which codes for the main regulator of lipolysis in adipocytes, responsible for releasing fatty acids from stored triglycerides. Mutations in this gene are directly related to several metabolic diseases [66]. In the Kalenjin’s highly differentiated genes, *MRAP2* codes for an accessory protein that modulates the activity of melanocortin receptors, in particular MC4R [67,68]. MC4R regulates physiological processes related to energy metabolism, having been associated with body mass index in African and European populations [69]. Mutations in *MRAP2* and *MC4R* cause obesity in both humans and mice [68,70,71]. *Mc4r* knocked-out (MC4RKO) mice are obese, but have a specific metabolic profile, with lower heart rate, lower lean body mass, lower muscle strength, lower bone density, and lower performance in endurance running [71].

Finally, muscle contraction in both skeletal and cardiac muscles depends on the calcium ion release from the sarcoplasmic reticulum into the cytoplasm [72]. Both Kalenjin and Oromo had many calcium-related enriched gene-sets (S4 Dataset), which were not observed for other ions like K^+^, Na^+^, Cl^-^ or Mg^2+^. Many genes discussed previously, like *WWOX*, *MIR6797*, *MRAP2*, *GSTO1*, and *RYR2* affect calcium homeostasis. Considering the system *GSTO1/RYR2*, high intrinsic aerobic exercise capacity in mice was associated with greater contraction amplitude in cardiomyocytes, an increased peak of calcium release, and increased expression of *RYR2* [73]. Besides, endurance training in rats induced higher contractibility and Ca^2+^ sensibility in cardiac muscle cells [74].

The fact that most genes discussed here have not been previously associated with endurance running may be due to differences in study design. Indeed, an important assumption of our study is that interpopulation comparisons may be more informative to explain the overrepresentation of East African ethnicities among elite runners [4]. On the other hand, former studies have relied on intrapopulation comparisons [11,75]. While case-control studies may help to validate or refute our findings, designing a proper case-control study is challenging [4]. Also, despite the overwhelming genetic diversity in Africa [76], most studies about genetics and athletic performance were either performed only in Eurasian populations [11,75], or used African populations to test for genetic associations originally discovered in Eurasians [6–8,75]. Remarkably, for many genes discussed here, a direct molecular interaction exists with genes previously associated with endurance running (e.g. *COL5A2* and *COL5A1*, *ARHGEF1* and *ACE*, and *WWOX* and *HIF1A*). This indicates that, while the same pathways are important for endurance running in different ethnicities, different genes may be more important in a population-specific context. *RYR2* and its regulator, *GSTO1*, being highly differentiated in Oromo and Kalenjin, respectively, further highlights this point. The overrepresentation of Eurasian populations in GWAS studies [77] may also explain, at least in part, why we found gene enrichment more often in Oromo, than in Kalenjin, both overall and for endurance relevant traits, given the much higher Eurasian ancestry in the former. Our results also reinforce the idea of endurance running as a complex and systemic activity [78]. Several genes (such as *WWOX*, *MRAP2*, *TRAM-AS1*, and *GSTO1*) seem to affect more than one trait, while all traits seem to be influenced by multiple genes, in a complex many-to-many relationship [79] that is also dependent on environmental and developmental processes. This view strengthens the criticism towards direct-to-consumer genetic tests to inform “genetic predisposition” for endurance sports, especially when used as a tool for prospecting children and young athletes for specific modalities [80].

Because we rely on a general measurement of population differentiation, our approach is also unable to test if endurance running evolved as an adaptation or should be seen as a by-product of neutral demographic processes. Another possibility is that adaptation to high-altitude favors some East African populations in endurance events, even though the direct relationship between being born in high-altitude and increased endurance performance is controversial [81]. We found only one gene, *RYR2*, in the Oromo list, that has been previously associated with high-altitude adaptation in studies involving East African (Amhara) populations [82], and another, *LIPE-AS1*, whose target, *LIPE*, was shown to affect survival rates in Drosophila exposed to low oxygen conditions [83]. However, while the Amhara are usually considered as a “model population” for studying high-altitude adaptation, they are not overrepresented among Ethiopian elite endurance athletes [2]. Finally, there were no enriched GWAS traits associated with high-altitude adaptation or hypoxia in neither Kalenjin nor Oromo. Taken together, it seems unlikely that high-altitude adaptation in East Africans is the major driver of endurance running success in these populations.

Finally, we would like to emphasize that genetic predisposition does not mean predestination, and success in sports should not be taken as a racial (or regional) stereotype (even if a putatively “positive” one). First, we must be very careful to avoid reinforcing the horrific racist ideas from the late 19^th^ century that, among other things, antagonized athleticism and intellectual ability [84]. A recent discussion in the US about racial stereotyping of black quarterbacks in American Football, for example, revealed that black athletes have been perceived as more “physical” and less “mental” than their white peers [85,86]. These associations are not only scientifically incorrect, but also ethically unacceptable. Second, as we have just emphasized, the genetics of complex traits is far from deterministic. Even though we restricted our analysis to genetic factors that may influence long-distance running, environmental, socio-cultural, and motivational factors must never be ignored [87,88]. Even if we understand that some populations have a higher frequency of alleles predisposing it to a specific phenotype, such as “long-distance running performance”, assuming that individuals from these populations adhere to the phenotype is an example of the ecological fallacy. Obviously, most Kalenjin and Oromo are not elite long-distance runners, and may never become one. Conversely, individuals from different ethnic backgrounds may become elite runners, such as the Olympic gold medalist Miruts Yifter, an Ethiopian long-distance athlete who was not from Oromo ethnicity, among a myriad of other examples. More than exploring the bases of long-distance running, this study illustrates the beauty of human genetic diversity and some of its fascinating physiological potentials.

## Materials and methods

### Basic assumptions and design

Our study assumes a “populational effect” for the dominance of some East African ethnicities in endurance running events [4]. That is, we expect that some of the more differentiated loci for Kalenjin and Oromo may be relevant to their endurance running success. As pointed out in the introduction, because we hypothesize that the alleles that predispose to endurance running should be common in the general population of these ethnic groups, there is no need to have athletes (or only athletes) among sampled individuals. This allows us to use data from public genomic datasets of genomic variation (see below). Again, our study is not the first to consider a phenotype as a populational characteristic. For example, studies about the genetic basis of altitude adaptation in humans often adopt this strategy, looking for peaks of genomic differentiation between closely related populations in high-altitude vs. lowland, regardless of possible intrapopulation individual differences in the response to high-altitude [12,13].

For estimating the peaks of genomic differentiation, we used the Population Branch Statistic (PBS) [12], in its standardized version, PBSn1, as described by Malaspinas *et al*. [14]. This statistic, which is based on the classical *F*_*ST*_ statistic, uses allele frequency data to estimate the degree of genetic differentiation specific to a population of interest, or focal population, in relation to two reference populations, generally one closely related to the focal population and another more distantly related [12,13]. It is important to note that, although widely used in adaptation studies, PBSn1 can be used to measure genetic differentiation regardless of the evolutionary processes causing differentiation (natural selection, genetic drift, admixture, etc.). This occurs because *F*_*ST*_, which is the basis of PBS (and PBSn1) calculation, is affected by several evolutionary processes [89]. When neutral evolutionary processes can be accounted for, PBS (and PBSn1) then becomes a measurement of genetic differentiation caused by adaptive processes (thus indicating natural selection) [90]. In this study, we do not make any assumptions about the evolutionary processes predisposing the Kalenjin and Oromo ethnic groups to endurance running dominance in sports events. In other words, we do not control for demographic history of neither population because we assume that both neutral and adaptive processes may have affected highly differentiated genomic regions associated with traits affecting endurance running.

Similarly, when the studied populations are admixed for different ancestries, differences in allele frequencies between ancestry components may affect genetic differentiation statistics. This must be corrected if the aim is to detect signals of natural selection [82,91]. However, when neutral processes are concerned, genomic admixture is a genuine process of population differentiation. In this study, we used different sets of closely and distantly related populations to account for admixture in the focal populations (see below for further details).

### Genomic datasets and population comparisons

We obtained curated genotyped SNP data made available by the African Genome Variation Project (AGVP), for 1,152,000 single nucleotide polymorphisms (SNPs) across the genome of 1,481 individuals from 18 ethnolinguistic groups from Sub-Saharan Africa using the Illumina Omni2.5 chip [76]. The data was subjected to the same quality control procedures employed by Gurdasani et al. [76], and only genotyped SNPs were considered (i.e. no imputation was performed). To access the diversity of other African and Eurasian populations used in the calculation of PBSn1, we also included equivalent data obtained from the 1000 Genomes Project database [92]. All data used in this study came from databases that were assembled respecting the ethical considerations elaborated by relevant research committees, both nationally (for the countries involved) and internationally.

Following demographic studies for Kenyan and Ethiopian athletes [2,3], we selected the Kalenjin (K, n = 100) and the Oromo (O, n = 26) as the focal populations in this study. These populations have differing levels of Eurasian ancestry (7.3–10.99% for Kalenjin, 43.62–50.82% for Oromo), and considerable diversity in their sub-Saharan African ancestry. Kalenjin has a greater contribution of Nilotic, followed by Horn of Africa and Southeast Bantu ancestries, while Oromo has a greater contribution of Horn of Africa ancestry [76]. We used two populations as “closely related”: the Luhya (L, n = 74, from Kenya) and the Amhara (A, n = 42, from Ethiopia), which were selected by being in the same countries as the focal populations (and theoretically, under the same socioeconomic policies), as well as for representing Bantu and Horn of Africa ancestries, respectively. For the distantly related population we built a metapopulation from a set of seven ethnic groups (Wolof, Mandinka, Jola, Fula, Ga-Adangbe, Yoruba, and Igbo, n = 618) representing Central and Western Africa (WA), in opposition to East Africa (Fig 1B). We also built a second metapopulation to be used as distantly related by combining six populations to represent Eurasia (EUR) from the 1000 Genomes Project (Individuals residing in the United States with European ancestry (CEU), Tuscan, Finnish, British, Iberian, and Individuals residing in the United States with Indian ancestry (GIH), n = 569). We used a Eurasian contrast as distantly related because the exclusive use of an African contrast could over-represent loci with high Eurasian ancestry in the focal populations due to Eurasian admixture. All procedures involving allele frequency calculation or population merging were performed in Plink 1.9 [93].

For the PBSn1 calculations, we used a focal population, a closely related and a distantly related population for all possible combinations, except for the two combinations that would include the Amhara and Eurasia. Those combinations were excluded because the Amhara have the highest level of Eurasian ancestry (47.82–54.70%) [76], and spurious results can emerge in PBSn1 when the two reference populations are closer to each other than to the focal population [14]. On the other hand, thanks to this level of mixing, the Amhara themselves act as good controls for Eurasian ancestry. Overall, we performed six comparisons, three for each focal population (focal population x closely related; distantly related): 1) K x L; WA, 2) K x L; EUR, 3) K x A; WA, 4) O x L; WA, 5) O x L; EUR, 6) O x A; WA. We estimated the PBSn1 values using in-house scripts in R [94]. We used sliding windows of 20 SNPs moving every five SNPs (thus, with overlapping of 15 SNPs), ensuring a homogeneous coverage of the genome and reducing the effect of individual SNPs. This resulted in a total 230,396 windows across the genome with a median size of 36,289 bp. We calculated the PBSn1 value for each SNP and then generated the average value of PBSn1 for the whole window. To check if the strategy of having multiple PBSn1 comparisons would effectively result in distinct ancestry components in different comparisons, we performed a discriminant analysis of principal components (DAPC) [95] for each of the six populations comparisons. This analysis was performed in the “adegenet” package [96] considering the populations as a priori clusters. We filtered the dataset for linkage disequilibrium and retained, based on principal component (PC) loadings, the first 400 PCs for the estimation of the discriminant function (S1 Fig).

When there is an adaptive hypothesis about the phenotype of interest, demographic simulations can be used to determine an empirical threshold above which the PBSn1 values are indicative of an adaptive process [13]. However, as previously stated, in this study we avoided any premise about evolutionary processes, assuming that purely demographic factors, such as drift and gene flow, may cause high PBSn1 values in loci associated with endurance running. In this case, it is impossible to establish an empirical significance value for PBSn1 from demographic simulations. Thus, following similar studies [13,97], we retained, from each comparison, the 0.1% windows with the highest PBSn1 values for further analysis.

### Annotation and statistical analysis

We used the Ensembl platform [98] to annotate the genes in the 5 Kb neighborhood of the SNP with the highest PBSn1 of each window in the top 0.1% PBSn1 values. We chose a short window size compared to other studies [82,99] to avoid including genes distantly linked to the highest peaks of genetic differentiation in the final gene list, as it could affect the enrichment analysis of gene-sets and endurance-relevant traits. Merging the lists from the three comparisons, we obtained a final list of highly differentiated genes for each focal population (S1 Dataset). We performed a gene-set analysis in the FUMA-GWAS platform [15], using the GENE2FUNC process, to determine which traits [16] and biological processes [100] were enriched for genes present in the original list (S2 and S4 Datasets) considering a *P*<0.05 after Benjamini-Hochberg False Discovery Ratio (FDR) correction. FUMA-GWAS performs the analysis using hypergeometric tests [15] to test for the enrichment of genes on our lists in gene-set from databases (GWAS-catalog [16] for traits and GO: Biological Process in MSigDB [100] for biological processes). The FDR correction is performed per data source of tested gene sets, using the number of gene sets in that data source (5,246 for GWAS-catalog and 7,658 for GO: Biological Process).

Our study design does not allow the specific detection of genes associated with endurance running success. However, we expect that genes associated with endurance running success will be among highly differentiated genomic regions in general. Conversely, many gene-sets with no biological relation with endurance running will show enrichment in the analysis.

As a “proof-of-concept”, we also reasoned that our approach should be able to detect genetic signals of other traits for which differences among populations are known. Ethiopian populations have lighter skin pigmentation compared to Kenyan populations. Therefore, the comparison (O x L; WA) should be enriched for gene-sets associated with skin pigmentation. Indeed, we found significant enrichment (*P* = 0.019) for “Skin pigmentation traits” (genes *BNC2* and *FANCA*). This example also illustrates the benefits of not controlling for admixture in some settings, since it is likely this difference would not emerge if only the sub-Saharan ancestry in the Oromo and Luhya were considered, given that it is probably associated with the large Eurasian ancestry in Ethiopian populations.

Following this, we tested if gene-sets associated with endurance-relevant traits would be overrepresented among enriched gene-sets. First, we selected from the full list of 5,246 traits from the GWAS Catalog database [16] all traits that could be relevant in endurance running. Four of the authors (A.L.S.Z., R.Q.C., R.L.M., and N.J.R.F) made the selection of these traits independently, based on a general literature for sports physiology (e.g. [101,102]). Traits selected at least twice composed the final list of endurance relevant traits (S3 Dataset). We compared the list of 628 endurance-relevant traits with the list of GWAS traits obtained from enriched gene-sets to test, for each population, if the number of matches was higher than expected by chance using Fisher’s exact test in R [94]. To further test the robustness of our results, we inverted the focal population and used the same comparisons and procedures to test if Luhya and Amhara were enriched for endurance-relevant traits. We note, however, that hypothesis does not require that these populations were not enriched for these traits, even though we would expect that these populations must either 1) show no significant enrichment, or 2) that the number of traits is lower compared to its national pair. Finally, the molecular functions of the five genes associated with the highest PBSn1 values from each comparison were also investigated for plausible biological associations with traits affecting endurance running.

### Sensitivity analyses

We also performed some sensitivity analyses to test the robustness of our results to parameter changes. We varied window size (original value, 20 SNPs with a 15 SNPs overlap; alternative value: 30 SNPs with a 20 SNPs overlap), the percentage of top-PBSn1 windows retained (original value 0.01%; alternative value 0.05%), and the reference list for endurance-relevant traits (original criteria: a trait had to be flagged by at least two of the four authors (2A) that checked the list of GWAS traits; alternative criteria: a trait had to be flagged by at least three authors (3A)). This alternative list of endurance-relevant traits is shown as S5 Dataset. We explored all combinations of parameters, and repeated the enrichment analysis of endurance-relevant traits for both Oromo and Kalenjin. The results of the sensitivity analyses can be found in S6 Dataset. Overall, the parameter changes had little impact on the analysis results. For Oromo, all parameter combinations resulted in statistically significant enrichment, with ORs varying from 1.67 (*P* = 3.0x10^-4^) to 3.09 (*P* = 0.008). For Kalenjin, six parameter combinations resulted in statistically significant enrichment, with ORs varying from 1.47 (*P* = 0.012) to 2.70 (*P* = 0.034). However, the two combinations including 30 SNPs windows and 0.1% of windows retained resulted in non-significant values (2A, OR = 1.84, *P* = 0.211; 3A, OR = 2.03 *P* = 0.280), possibly because the number of total sets (and hence the number of endurance-related sets) was too small, reducing the statistical power of Fisher’s exact test. All endurance-related gene-sets detected in the sensitivity analyses are shown in S7 Dataset.

## Supporting information

S1 Fig Discriminant analysis of principal components (DAPC) for the six population comparisons performed. (DOCX)

S1 Table Enriched gene-sets for “endurance-relevant” traits in GWAS Catalog (P<0.05 after FDR correction) for both Kalenjin and Oromo. (DOCX)

S1 Text Further information about the studied populations. (DOCX)

S1 Dataset List of the genes associated with the 0.1% most differentiated windows, by comparison, for Kalenjin and Oromo. (XLSX)

S2 Dataset List of all enriched gene-sets (including those not endurance-relevant), in GWAS Catalog, considering all retained genes for Kalenjin and Oromo. (XLSX)

S3 Dataset List of all gene-sets in GWAS Catalog selected as “endurance-relevant”, according with the literature, by at least 2 authors (see Material and Methods for details). (XLSX)

S4 Dataset List of all enriched gene-sets (including those not endurance-relevant), in GO biological processes, considering all retained genes for Kalenjin and Oromo. (XLSX)

S5 Dataset List of all gene-sets in GWAS Catalog selected as “endurance-relevant”, according with the literature, by at least 3 authors (see Material and Methods for details). (XLSX)

S6 Dataset Results for the sensitivity analyses (see Material and Methods for details). (XLSX)

S7 Dataset Enriched gene-sets for “endurance-relevant” traits in GWAS Catalog (P<0.05 after FDR correction) for both Kalenjin and Oromo, considering the parameters used in the sensitivity analyses. (XLSX)
