# Supplementary material for: Genetic differentiation in East African ethnicities and its relationship with endurance running success
Source: PLoS One. 2022 May 19;17(5):e0265625. doi: 10.1371/journal.pone.0265625 (PMC9119534; doi:10.1371/journal.pone.0265625)
Supplement: S1 Fig — (DOCX) [file pone.0265625.s001.docx]

S1 Fig. Discriminant analysis of principal components (DAPC) for the six population comparisons performed. Note that different comparisons result in a different structure for the focal populations. For example, in comparisons in which EUR is the distantly related population, the focal population is closer to the remaining African populations, as expected.
