# Supplementary material for: Genetic differentiation in East African ethnicities and its relationship with endurance running success
Source: PLoS One. 2022 May 19;17(5):e0265625. doi: 10.1371/journal.pone.0265625 (PMC9119534; doi:10.1371/journal.pone.0265625)
Supplement: S1 Table — (DOCX) [file pone.0265625.s002.docx]

Table S1 – Enriched gene-sets for “endurance-relevant” traits in GWAS Catalog (P<0.05 after FDR correction) for both Kalenjin and Oromo.

| GWAS catalog gene-set | Genes in set (total) | Genes in set | | *P*-value | |
| --- | --- | --- | --- | --- | --- |
|  |  | Kalenjin | Oromo | Kalenjin | Oromo |
| Diastolic blood pressure | 648 | - | 22 | - | <0.001 |
| Hand grip strength | 156 | - | 9 | - | <0.001 |
| Pulse pressure | 690 | - | 18 | - | <0.001 |
| Heel bone mineral density | 834 | 19 | 15 | <0.001 | 0.032 |
| Systolic blood pressure | 783 | - | 19 | - | 0.001 |
| Body mass index | 1197 | - | 24 | - | 0.001 |
| Waist circumference | 73 | 4 | 6 | 0.037 | 0.002 |
| Height | 529 | 10 | 14 | 0.044 | 0.003 |
| Lung function (FVC) | 178 | - | 8 | - | 0.004 |
| Waist-to-hip ratio adjusted for BMI | 350 | 9 | 11 | 0.025 | 0.004 |
| Lung function (FEV1) | 65 | - | 5 | - | 0.006 |
| Peak expiratory flow | 115 | - | 6 | - | 0.009 |
| Waist circumference adjusted for BMI in active individuals | 83 | - | 5 | - | 0.014 |
| Body fat distribution (arm fat ratio) | 129 | - | 6 | - | 0.014 |
| Atrial fibrillation | 238 | - | 8 | - | 0.014 |
| Waist circumference adjusted for BMI (joint analysis main effects and physical activity interaction) | 85 | - | 5 | - | 0.014 |
| Lung function (FEV1/FVC) | 184 | 6 | 7 | 0.034 | 0.015 |
| Bone mineral density (hip) | 55 | - | 4 | - | 0.021 |
| Hypertension | 100 | - | 5 | - | 0.023 |
| Hip circumference adjusted for BMI | 101 | - | 5 | - | 0.024 |
| Waist-hip ratio | 60 | - | 4 | - | 0.025 |
| Lean body mass | 7 | - | 2 | - | 0.028 |
| Hip shape (DXA scan) | 6 | 2 | - | 0.031 | - |
| Subcutaneous adipose tissue | 62 | 4 | - | 0.031 | - |
| Vigorous physical activity | 8 |  | 2 |  | 0.032 |
| FEV1 | 175 | 6 | 6 | 0.033 | 0.036 |
| LDL cholesterol | 181 | 6 | - | 0.033 | - |
| Waist-to-hip ratio adjusted for body mass index | 114 | - | 5 | - | 0.034 |
| Glucose homeostasis traits | 73 | 4 | - | 0.037 | - |
| Hip circumference | 73 | - | 4 | - | 0.039 |
| Waist circumference adjusted for body mass index | 126 | - | 5 | - | 0.044 |
