# Supplementary material for: Genetic differentiation in East African ethnicities and its relationship with endurance running success
Source: PLoS One. 2022 May 19;17(5):e0265625. doi: 10.1371/journal.pone.0265625 (PMC9119534; doi:10.1371/journal.pone.0265625)
Supplement: S1 Text — (DOCX) [file pone.0265625.s003.docx]

**Genetic Differentiation in East African Ethnicities and Its Relationship with Endurance Running Success**

André L. S. Zani, Mateus H. Gouveia, Marla M. Aquino, Rodrigo Quevedo, Rodrigo L. Menezes, Charles Rotimi, Gerald O. Lwande, Collins Ouma, Ephrem Mekonnen, and Nelson J. R. Fagundes

Nelson J. R. Fagundes

Email: [nelson.fagundes@ufrgs.br](mailto:nelson.fagundes@ufrgs.br)

S1 Text

Ethiopia and Kenya are countries located in sub-Saharan Africa, one of the world's most ethnically, culturally and geographically diverse regions. Ethiopia has 87 different indigenous languages [1] spoken across 85 distinct ethnicities [2] and shows a complex mosaic of genomic ancestries [3] resulting from an interplay between geographical and cultural factors [4]. Together with its geographic position and history, Ethiopian populations have caught the attention of geneticists since earlier studies of human genetic variation using blood groups and classical markers [5, 6], and has been fundamental to test hypotheses on human migration routes of both the Out of Africa’ and ‘Into Africa’ models [7, 8].

More recently, Ethiopian populations have been studied to unravel the genetic signals of adaptation to living in high-altitude [9-11] and milk drinking [12-14], while other studies have been conducted to explore the diversity of drug metabolizing enzymes [15-17] and, more recently, to analyze the genetic epidemiology of susceptibility to diseases [18, 19]. The identification of high genetic diversity and novel variants in Ethiopian populations, coupled with their dominance in endurance running, especially from people of the Oromia region [20], has also led to the inclusion of Ethiopian populations in studies of the genetics of elite athletes [21-23].

Similarly, Kenya is multicultural and ethnically diverse, with 61 different indigenous languages [1] spoken by 41 ethnicities [24] whose genomic is a mosaic of Bantu, Nilotic, Horn of Africa and Eurasian ancestries [25]. The majority of the studies conducted in the western Kenya region have focused on the genetic basis of susceptibility to infectious diseases such as malaria [26-28], tuberculosis [29, 30] as well as pharmacogenomics and drug resistance patterns for these infectious diseases [28, 31, 32]. Over the last few years, new studies have begun focusing on genetic predisposition to non-infectious diseases such as cancer and cardiovascular diseases [33-37]. These studies had led to the question of what genetic lessons could be learnt from high-altitude living Kenyans within the Rift Valley region who have been shown to perform outstandingly well in global endurance athletics competitions [38-40].

The majority of these globally renowned athletes come from the Kalenjin community [41, 42], who are highland Nilotes and include eight subtribes (Nandi, Keiyo, Kipsigis, Sabaot, Marakwet, Tugen, Injems and Terik). Geographically, they live adjacent to the Luos, who are river lake Nilotes living around the Lake Victoria basin, as well as to the Luhya, who are of predominantly Bantu ancestry [43]. Even though the Nandi is the most famous Kalenjin subtribe associated with endurance running [42], the Sebei, a Ugandan Kalenjin speaking population living around the Mount Elgon, in the border of Kenya, have been gaining much success in endurance running competitions [44].

**References**

1. Eberhard DM, Simons GF, Fennig CD. Ethnologue: Languages of the World. Dallas, Texas: SIL International; 2021. Available: http://www.ethnologue.com.

2. Central Statistics Agency. Population and Housing Census 2007. Available: https://www.statsethiopia.gov.et/wp-content/uploads/2019/06/National_Statistical.pdf.

3. Hellenthal G, Bird N, Morris S. Structure and ancestry patterns of Ethiopians in genome-wide autosomal DNA. Hum Mol Genet. 2021;30: R42–R48.

4. López S, Tarekegn A, Band G, van Dorp L, Bird N, Morris S, et al. Evidence of the interplay of genetics and culture in Ethiopia. Nat Commun. 2021;12: 3581.

5. Mourant AE, Kopeć AC, Ikin EW, Lehmann H, Bowen-Simpkins P, Fergusson IL, et al. The blood groups and haemoglobins of the Kunama and Baria of Eritrea, Ethiopia. Ann Hum Biol. 1974;1: 383–392.

6. Harrison GA. Genetic and anthropological studies in the human adaptability section of the International Biological Programme. Philos Trans R Soc Lond B Biol Sci. 1976;274: 437–445.

7. Pagani L, Schiffels S, Gurdasani D, Danecek P, Scally A, Chen Y, et al. Tracing the route of modern humans out of Africa by using 225 human genome sequences from Ethiopians and Egyptians. Am J Hum Genet. 2015;96: 986–991.

8. Gallego Llorente M, Jones ER, Eriksson A, Siska V, Arthur KW, Arthur JW, et al. Ancient Ethiopian genome reveals extensive Eurasian admixture throughout the African continent. Science. 2015;350: 820–822.

9. Huerta-Sánchez E, Degiorgio M, Pagani L, Tarekegn A, Ekong R, Antao T, et al. Genetic signatures reveal high-altitude adaptation in a set of ethiopian populations. Mol Biol Evol. 2013;30: 1877–1888.

10. Stobdan T, Zhou D, Ao-Ieong E, Ortiz D, Ronen R, Hartley I, et al. Endothelin receptor B, a candidate gene from human studies at high altitude, improves cardiac tolerance to hypoxia in genetically engineered heterozygote mice. Proc Natl Acad Sci U S A. 2015;112: 10425–10430.

11. Simonson TS. Altitude adaptation: A glimpse through various lenses. High Alt Med Biol. 2015;16: 125–137.

12. Liebert A, López S, Jones BL, Montalva N, Gerbault P, Lau W, et al. World-wide distributions of lactase persistence alleles and the complex effects of recombination and selection. Hum Genet. 2017;136: 1445–1453.

13. Jones BL, Raga TO, Liebert A, Zmarz P, Bekele E, Danielsen ET, et al. Diversity of lactase persistence alleles in Ethiopia: signature of a soft selective sweep. Am J Hum Genet. 2013;93: 538–544.

14. Ingram CJE, Raga TO, Tarekegn A, Browning SL, Elamin MF, Bekele E, et al. Multiple rare variants as a cause of a common phenotype: several different lactase persistence associated alleles in a single ethnic group. J Mol Evol. 2009;69: 579–588.

15. Creemer OJ, Ansari-Pour N, Ekong R, Tarekegn A, Plaster C, Bains RK, et al. Contrasting exome constancy and regulatory region variation in the gene encoding CYP3A4: an examination of the extent and potential implications. Pharmacogenet Genomics. 2016;26: 255–270.

16. Browning SL, Tarekegn A, Bekele E, Bradman N, Thomas MG. CYP1A2 is more variable than previously thought: a genomic biography of the gene behind the human drug-metabolizing enzyme. Pharmacogenet Genomics. 2010;20: 647–664.

17. Sim S, Risinger C, Dahl M, Aklillu E, Christensen M, Bertilsson L, et al. A common novel CYP2C19 gene variant causes ultrarapid drug metabolism relevant for the drug response to proton pump inhibitors and antidepressants. Clin Pharmacol Ther. 2006;79: 103–113.

18. Mekonnen E, Bekele E, Stein CM. Novel polymorphisms in TICAM2 and NOD1 associated with tuberculosis progression phenotypes in Ethiopian populations. Glob Health Epidemiol Genom. 2018;3. doi:10.1017/gheg.2017.17

19. Mekonnen E, Bekele E. An ancestral human genetic variant linked to an ancient disease: A novel association of FMO2 polymorphisms with tuberculosis (TB) in Ethiopian populations provides new insight into the differential ethno-geographic distribution of FMO2*1. PLoS One. 2017;12: e0184931.

20. Scott RA, Georgiades E, Wilson RH, Goodwin WH, Wolde B, Pitsiladis YP. Demographic characteristics of elite Ethiopian endurance runners. Med Sci Sports Exerc. 2003;35: 1727–1732.

21. Rankinen T, Fuku N, Wolfarth B, Wang G, Sarzynski MA, Alexeev DG, et al. No evidence of a common DNA variant profile specific to world class endurance athletes. PLoS One. 2016;11: e0147330.

22. Ash GI, Scott RA, Deason M, Dawson TA, Wolde B, Bekele Z, et al. No association between ACE gene variation and endurance athlete status in Ethiopians. Med Sci Sports Exerc. 2011;43: 590–597.

23. Scott RA, Wilson RH, Goodwin WH, Moran CN, Georgiades E, Wolde B, et al. Mitochondrial DNA lineages of elite Ethiopian athletes. Comp Biochem Physiol B Biochem Mol Biol. 2005;140: 497–503.

24. Kenya National Bureau of Statistics. 2019 Kenya Population and Housing Census Volume IV: Distribution of Population by Socio-Economic Characteristics. Available: https://www.knbs.or.ke/?wpdmpro=2019-kenya-population-and-housing-census-volume-iv-distribution-of-population-by-socio-economic-characteristics

25. Gurdasani D, Carstensen T, Tekola-Ayele F, Pagani L, Tachmazidou I, Hatzikotoulas K, et al. The African Genome Variation Project shapes medical genetics in Africa. Nature. 2015;517: 327–332.

26. Ndila CM, Uyoga S, Macharia AW, Nyutu G, Peshu N, Ojal J, et al. Human candidate gene polymorphisms and risk of severe malaria in children in Kilifi, Kenya: a case-control association study. Lancet Haematol. 2018;5: e333–e345.

27. Williams TN, Mwangi TW, Roberts DJ, Alexander ND, Weatherall DJ, Wambua S, et al. An immune basis for malaria protection by the sickle cell trait. PLoS Med. 2005;2: e128.

28. Hemming-Schroeder E, Umukoro E, Lo E, Fung B, Tomás-Domingo P, Zhou G, et al. Impacts of antimalarial drugs on Plasmodium falciparum drug resistance markers, western Kenya, 2003-2015. Am J Trop Med Hyg. 2018;98: 692–699.

29. Nyamogoba H, Mbuthia G. Gender-age distribution of tuberculosis among suspected tuberculosis cases in western Kenya. Med Sci (Turkey). 2018; 1.

30. Mbugi EV, Katale BZ, Streicher EM, Keyyu JD, Kendall SL, Dockrell HM, et al. Mapping of Mycobacterium tuberculosis complex genetic diversity profiles in Tanzania and other African countries. PLoS One. 2016;11: e0154571.

31. Hu L, Hogan JW, Mwangi AW, Siika A. Modeling the causal effect of treatment initiation time on survival: Application to HIV/TB co-infection. Biometrics. 2018;74: 703–713.

32. Onyango DO, Yuen CM, Cain KP, Ngari F, Masini EO, Borgdorff MW. Reduction of HIV-associated excess mortality by antiretroviral treatment among tuberculosis patients in Kenya. PLoS One. 2017;12: e0188235.

33. Manji I, Pastakia SD, Do AN, Ouma MN, Schellhase E, Karwa R, et al. Performance outcomes of a pharmacist-managed anticoagulation clinic in the rural, resource-constrained setting of Eldoret, Kenya: Performance outcomes of a pharmacist-managed anticoagulation clinic. J Thromb Haemost. 2011;9: 2215–2220.

34. Vedanthan R, Kamano JH, Naanyu V, Delong AK, Were MC, Finkelstein EA, et al. Optimizing linkage and retention to hypertension care in rural Kenya (LARK hypertension study): study protocol for a randomized controlled trial. Trials. 2014;15: 143.

35. Bloomfield GS, DeLong AK, Akwanalo CO, Hogan JW, Carter EJ, Aswa DF, et al. Markers of atherosclerosis, clinical characteristics, and treatment patterns in heart failure: A case-control study of middle-aged adult heart failure patients in rural Kenya. Glob Heart. 2016;11: 97.

36. Pastakia SD, Ali SM, Kamano JH, Akwanalo CO, Ndege SK, Buckwalter VL, et al. Screening for diabetes and hypertension in a rural low income setting in western Kenya utilizing home-based and community-based strategies. Global Health. 2013;9: 21.

37. Temu TM, Lane KA, Shen C, Ng’ang’a L, Akwanalo CO, Chen P-S, et al. Clinical characteristics and 12-month outcomes of patients with valvular and non-valvular atrial fibrillation in Kenya. PLoS One. 2017;12: e0185204.

38. Haile DW, Durussel J, Mekonen W, Ongaro N, Anjila E, Mooses M, et al. Effects of EPO on blood parameters and running performance in Kenyan athletes. Med Sci Sports Exerc. 2019;51: 299–307.

39. Scott RA, Pitsiladis YP. Genotypes and distance running: Clues from Africa. Sport Med. 2007;37: 424–427.

40. Scott RA, Moran C, Wilson RH, Goodwin WH, Pitsiladis YP. Genetic influence on East African running success. Equine Comp Exerc Physiol. 2004;1: 273–280.

41. Pitsiladis YP, Onywera VO, Geogiades E, O’Connell W, Boit MK. The dominance of Kenyans in distance running. Equine Comp Exerc Physiol. 2004;1: 285–291.

42. Onywera VO, Scott RA, Boit MK, Pitsiladis YP. Demographic characteristics of elite Kenyan endurance runners. J Sports Sci. 2006;24: 415–422.

43. Mulama DH, Bailey JA, Foley J, Chelimo K, Ouma C, Jura WGZO, et al. Sickle cell trait is not associated with endemic Burkitt lymphoma: an ethnicity and malaria endemicity-matched case-control study suggests factors controlling EBV may serve as a predictive biomarker for this pediatric cancer: Sickle cell trait and EBV loads in endemic Burkitt lymphoma. Int J Cancer. 2014;134: 645–653.

44. Epstein D. The sports gene: Inside the science of extraordinary athletic performance. Portfolio; 2014.
